# Exploring New Alleles Involved in Tomato Fruit Quality in an Introgression Line Library of *Solanum pimpinellifolium*

**DOI:** 10.3389/fpls.2016.01172

**Published:** 2016-08-17

**Authors:** Walter Barrantes, Gloria López-Casado, Santiago García-Martínez, Aranzazu Alonso, Fernando Rubio, Juan J. Ruiz, Rafael Fernández-Muñoz, Antonio Granell, Antonio J. Monforte

**Affiliations:** ^1^Instituto de Biología Molecular y Celular de Plantas, Consejo Superior de Investigaciones Científicas, Polytechnic University of ValenciaValencia, Spain; ^2^Estación Experimental Agrícola Fabio Baudrit Moreno, Universidad de Costa RicaAlajuela, Costa Rica; ^3^Instituto de Hortofruticultura Subtropical y Mediterránea “La Mayora”, Consejo Superior de Investigaciones Científicas, University of MalagaAlgarrobo-Costa, Spain; ^4^Departamento de Biología Aplicada, Escuela Politécnica Superior de Orihuela, Universidad Miguel HernándezOrihuela, Spain

**Keywords:** quantitative trait loci (QTL), germplasm, wild species, mapping, genotype by environment interaction

## Abstract

We have studied a genomic library of introgression lines from the *Solanum pimpinellifolium* accession TO-937 into the genetic background of the “Moneymaker” cultivar in order to evaluate the accession’s breeding potential. Overall, no deleterious phenotypes were observed, and the plants and fruits were phenotypically very similar to those of “Moneymaker,” which confirms the feasibility of translating the current results into elite breeding programs. We identified chromosomal regions associated with traits that were both vegetative (plant vigor, trichome density) and fruit-related (morphology, organoleptic quality, color). A trichome-density locus was mapped on chromosome 10 that had not previously been associated with insect resistance, which indicates that the increment of trichomes by itself does not confer resistance. A large number of quantitative trait loci (QTLs) have been identified for fruit weight. Interestingly, fruit weight QTLs on chromosomes 1 and 10 showed a magnitude effect similar to that of QTLs previously defined as important in domestication and diversification. Low variability was observed for fruit-shape-related traits. We were, however, able to identify a QTL for shoulder height, although the effects were quite low, thus demonstrating the suitability of the current population for QTL detection. Regarding organoleptic traits, consistent QTLs were detected for soluble solid content (SSC). Interestingly, QTLs on chromosomes 2 and 9 increased SSC but did not affect fruit weight, making them quite promising for introduction in modern cultivars. Three ILs with introgressions on chromosomes 1, 2, and 10 increased the internal fruit color, making them candidates for increasing the color of modern cultivars. Comparing the QTL detection between this IL population and a recombinant inbred line population from the same cross, we found that QTL stability across generations depended on the trait, as it was very high for fruit weight but low for organoleptic traits. This difference in QTL stability may be due to a predominant additive gene action for QTLs involved in fruit weight, whereas epistatic and genetic background interactions are most likely important for the other traits.

## Introduction

Cultivated tomato, *Solanum lycopersicum* L., has undergone two domestication steps during its history (Blanca et al., 2012, 2015). The first domestication occurred early on in Ecuador and northern Peru, and was most likely carried out by ancient farmers on *Solanum pimpinellifolium* L. and/or *S. lycopersicum* var. *cerasiforme*. The second step most likely took place in Mesoamerica due to migrated pre-domesticated tomatoes. Spanish conquistadors brought the tomato from Mesoamerica to Europe, and from there it was spread all around the world. One consequence of this process was dramatic genetic erosion, caused especially by the bottleneck during the migration from the Andean regions to Mesoamerica. Early research (Williams and Clair, 1993) showed a much higher diversity in Andean *S. pimpinellifolium* and *S. lycopersicum* var. *cerasiforme* populations than among Mesoamerican cultivars, and this has been supported by recent high density single nucleotide polymorphism (SNP) variability analysis (Blanca et al., 2015). Therefore, accessions originating anywhere from the Andes to Mesoamerica will most certainly prove to be an important source of useful genetic diversity for tomato breeding.

After the introduction of tomato into Europe, intense breeding efforts were carried out in order to increase yield, adaptation, stability, and disease resistance (Bai and Lindhout, 2007). Despite these common objectives, the breeding goals changed over time due to the requirements of specific markets and uses. During the 1970 and 1980s, one of the most important breeding objectives, especially for fresh-market tomatoes, was to increase yield and shelf life. Both breeding objectives resulted in improved external quality, although at the expense of internal fruit quality. During the following decade, taste became the main breeding objective. Sugars, acids and more than 30 volatile compounds are known to influence tomato flavor (Tieman et al., 2012; Rambla et al., 2014). Organoleptic quality is a very complex trait as it depends on the evolving preferences of the market. Even so, significant improvement in tomato flavor seems possible by increasing the fruit sugar and acid contents and by modifying the balance between the two (Stevens et al., 1977). Wild species have mostly been used to introduce resistance genes, thus increasing the genetic diversity of modern cultivars compared to vintage cultivars (Sim et al., 2009, 2011, 2012).

The complex polygenic control of tomato fruit quality traits involves multiple quantitative trait loci (QTLs; Labate et al., 2007). Interestingly, favorable effects on fruit quality have been identified in wild species, such as *S. pimpinellifolium, Solanum pennellii, Solanum cheesmaniae*, and *Solanum habrochaites* (Eshed and Zamir, 1995; Bernacchi et al., 1998; Monforte et al., 2001; Fulton et al., 2002; Lippman et al., 2007), even though the fruits of these species are not usually consumed by humans. The exploitation of these QTLs in practical breeding has been very limited because of the inherent difficulties in implementing marker-assisted selection (MAS) for QTLs (Collard and Mackill, 2008) in addition to deleterious linkage drag. Nevertheless, a few successful studies have been reported (Gur and Zamir, 2004).

Both advanced backcross and introgression lines (ILs) may be used to facilitate the incorporation of genetic variability from wild species (Eshed and Zamir, 1995; Tanksley et al., 1996). ILs are developed by MAS and contain a unique chromosome fragment from a donor genotype (usually a wild species or unadapted germplasm) in a uniform elite genetic background. These collections are also called “genomic libraries of ILs” when the whole genome of the donor genotype is represented among the introgressions.

In tomato, ILs have been developed from *S. pennellii* LA0716 (Eshed and Zamir, 1994), *S. habrochaites* LA1777 (Monforte and Tanksley, 2000a), *Solanum lycopersicoides* LA 2951 (Chetelat and Meglic, 2000; Canady et al., 2005), *S. habrochaites* LA0407 (Francis et al., 2001) and *S. habrochaites* LYC4 (Finkers et al., 2007). In addition, a small number of ILs have been developed for the *S. pimpinellifolium* accessions LA1589 (Tanksley et al., 1996; Bernacchi et al., 1998) and LA2093 (Kinkade and Foolad, 2013). IL collections are extremely useful for identifying QTLs (Eshed and Zamir, 1995; Rousseaux et al., 2005), verifying QTL effects (Tanksley et al., 1996), studying QTL x environmental, QTL x genetic background and QTL x QTL interactions (Monforte et al., 2001), QTL fine mapping (Eshed and Zamir, 1996; Ku et al., 2000; Monforte and Tanksley, 2000b; Ashrafi et al., 2012) and introducing new genetic variability from wild species into elite germplasm (Tanksley and McCouch, 1997; Zamir, 2001; Gur and Zamir, 2004). IL analysis is also a powerful tool for genomics research, as it facilitates the study of the genetic basis of metabolome (Schauer et al., 2006), transcriptome and its correlation with metabolome (Lee et al., 2012), enzyme activity (Steinhauser et al., 2011) and QTL cloning (Frary et al., 2000; Fridman et al., 2000; Liu et al., 2002). The most widely used IL collection is the *S. pennellii* LA 0716 collection, which has facilitated the identification of more than 2,700 QTLs involved in agronomical important characters (Lippman et al., 2007).

The development of IL collections has traditionally required intense effort spanning several years (Eshed and Zamir, 1994; Eduardo et al., 2005). Barrantes et al. (2014) applied high-throughput genotyping in an IL breeding program, demonstrating that IL libraries can now be produced with far less effort at a lower cost. The IL population thus produced was derived from a cross between the cultivar Moneymaker (*S. lycopersicum*) and the *S. pimpinellifolium* accession TO-937. This accession is from Peru, i.e., the region where the tomato most likely underwent its first domestication step. It is therefore a quite suitable accession for introducing new genetic variability into the cultivated tomato genetic pool. Previous works have demonstrated that TO-937 harbors genetic variability that is of interest for breeding purposes, such as enhancing ascorbic acid (Lima-Silva et al., 2012), sugar, organic acid and carotenoid fruit content (Capel et al., 2015), as well as modifying aroma volatile compounds (Rambla et al., 2014) and resistance to pests (Fernández-Muñoz et al., 2000; Silva et al., 2014). Furthermore, populations derived from this accession were used to map the *Uniform ripening* (*U*) locus (Powell et al., 2012). In the current report, we present a thorough phenotypic characterization of this IL library, focusing mainly on fruit traits and the characterization of QTLs involved in fruit quality as the first step in introducing new genetic variability into the elite tomato gene pool.

## Materials and Methods

### Plant Material

A complete genomic library of 54 ILs derived from a cross between the wild *S. pimpinellifolium* (SP) accession TO-937 as a donor parent, obtained from the Instituto de Hortofruticultura Subtropical y Mediterránea “La Mayora” (IHSM-UMA-CSIC) germplasm bank, and the cultivar “Moneymaker” (*S. lycopersicum*) as recurrent parent (hereafter referred to as MM, Barrantes et al., 2014) were studied in the current report. In brief, each IL contains an average of 3.7% of the SP genome (range: 0.5–7.7%), altogether covering 98.8% of the donor parent genome, with an average introgression size of 25 Mb (ranging from 0.7 to 75 Mb). IL evaluation was performed during the spring-summer of 2013 at three locations in Spain: Alginet, Valencia (Agricultural Cooperative Alginet Coagri), School of Engineering of Orihuela, Alicante (Miguel Hernández University) and Algarrobo-Costa, Málaga (IHSM-UMA-CSIC). All three locations are on the Mediterranean coast of Spain, and, therefore, have similar weather conditions. ILs were grown in plastic greenhouses following a randomized complete block design with eight blocks, each containing one replicate per IL and six replicates of MM. In the first through seventh blocks, each replicate had a single plant, whereas in the eighth block each replicate consisted of three plants. The eighth block was used to better distinguish categorical traits between the ILs and MM.

### Phenotypic Analysis

Traits were classified into two categories: descriptive and quantitative traits. Descriptive traits were only evaluated in block 8, as each replicate consisted of three plants, which made it easier to observe the differences between the ILs and MM categorically. This group of traits included: vigor (VIG), as the height of the plant at first fruit set expressed in cm; purple (PURP), as the presence or absence of anthocyanin coloration in branches and stems; trichomes (TRI), visually observed long trichome density on stems with a scale of: 0 = absence, 1 = low, 2 = medium, and 3 = high; and earliness (EAR), as days from transplanting to first ripe fruit and presence/absence of dark green shoulder (GS) on breaker fruit. Quantitative fruit traits were evaluated on four fruits harvested at light-red stage, selected from a large sample of fruits for being the most representative as regards homogeneity in maturity and size. A single sample per plant was obtained for blocks 1–7, whereas a pooled sample of three plants was obtained in block 8. Each fruit was weighted (FW in grams) and the external color (EC) was recorded at three points in the equatorial region of the tomato fruit using a Minolta Chroma Meter model CR-400 (Konica Minolta, Inc., Tokyo, Japan), applying the CIE Lab color space, where higher +a^∗^ indicates red and lower -a^∗^ indicates green, whereas higher b^∗^ indicates yellow and lower b^∗^ green. The color space is three-dimensional, where the third axis, L^∗^, represents black to white and the a^∗^-b^∗^ plane may be visualized as a color wheel that is lighter or darker depending on the level of L^∗^. Lower L^∗^ values represent a darker color. Chroma (C^∗^), a measure of color saturation, was calculated using the formula: (a^∗2^+b^∗2^)^1/2^. Hue-angle (H), in degrees, is the measurement of an object’s color in the a^∗^-b^∗^ plane and was calculated as (180/p)_∗_cos^-1^(a^∗^/C^∗^) for the positive values of b^∗^ obtained. Perception of hue angle differences depends on the chroma, with the differences being more detectable at higher chroma (Sacks and Francis, 2001). After longitudinal cutting, fruits were scanned at 300 dots per inch (dpi). The images were saved as jpeg files and imported into Tomato Analyzer 3.0 software for automated phenotypic analysis^1^. Fruit morphology descriptors were following Brewer et al. (2006): maximum diameter (FD, in cm), max length (FL, in cm) fruit shape: fruit shape index (FS), fruit shape circular (CIR), shoulder height (PSH, that is a measurement of the indentation of peduncle scar at the proximal end of the fruit). For internal color (IC), the Tomato Analyzer color module was calibrated with a scanned X-Rite Color Checker card. Images were previously processed with Photoshop CS5 v. 12. (Adobe Systems Incorporated, San Jose, CA, USA) in order to save an image of the same fragment of pericarp tissue in each fruit. Finally, CIELab color parameters were obtained using Tomato Analyzer (Darrigues et al., 2008). The organoleptic traits, such as soluble solid content (SSC), pH (PH), and titratable acidity (TA) were analyzed from tomato pericarp tissue from four fruits ground and stored at -20°C. Samples were thawed and centrifuged at 3500 rpm for 10 min, an aliquot of supernatant was used for measuring SSC (expressed in °Brix) using a digital refractometer (Atago CO LTD, Tokyo, Japan) and PH and TA (expressed in percentage of citric acid) were determined from 1 ml of the supernatant homogenized juice with the electronic analyzer PH-Matic23 (CRISON, Barcelona, Spain).

### Statistical Analyses

For each trait, the genetic (G), location (L) and interaction (G-x-L) effects were estimated by two-way ANOVA. Heritability was estimated by one-way ANOVA in each locality (G-x-L was significant for nearly all traits, see below) as *h^2^= V_g/_V_t_* (where *V_g_* represents genetic variance, estimated as the variance among genotypes, and *V_t_* represents total variance). Pearson’s correlation coefficients among traits were calculated in each location. IL and control MM means were compared by a Dunnet’s test at *p* < 0.05. Only ILs that were significantly different from MM in at least two locations were considered for QTL assignment. QTLs were mapped in the chromosome regions that were covered by the TO-937 introgressions in the ILs that showed significant effects on the trait under study. In those cases where the means of two ILs with overlapping introgressions were significantly different from MM, a contrast test was performed between those ILs. When IL means were not different, the QTL was assumed to be located in the overlapping regions; when the means were different, two QTLs were assumed. All statistical analyses were performed with JMP v. 11 (SAS Institute, Cary, NC, USA).

## Results

### Recurrent Parent (MM)

The trait means and standard deviations of parental MM at the three locations are presented in Supplementary Table S1. No significant differences in FW were found between locations, with the average being 98.1 g, whereas other fruit dimension traits such as FL and FD showed significant differences (*p* ≤ 0.01), with higher values in Málaga. Shape-related traits showed statistically significant differences among locations (*p* ≤ 0.01), except for FS. Significant differences for organoleptic traits (SSC, PH, and TA) and color traits (IC and EC) were also found among locations.

### Introgression Lines and QTL Mapping

In general, the phenotypes of both plant and fruit were very similar to the phenotypes of the recurrent MM. Only in very few cases did we observe extreme phenotypes. A thorough description of the phenotypes and the QTL mapping can be found below. In Supplementary Table S2, a summary of the comparison of each IL with MM across all locations is also shown.

#### Descriptive Traits

During the cultivation of the IL collection, several phenotypic characteristics that were consistent in at least two locations were clearly observed (Supplementary Figure S1). ILs SP_2-4 and SP_2-5 showed fast vegetative growth, defining a locus for plant vigor (*vig2.1*). SP_3-1, on the other hand, suffered such a drastic reduction of plant growth that it did not set fruits in two locations, which, in turn, permitted to define another locus (*vig3.1*). Furthermore, SP_5-2 and SP_5-3 displayed purple shoots, probably due to anthocyanin accumulation (*purp5.1*). SP_10-3, meanwhile, had denser trichomes (*tri10.1*) than MM. IL_2-5 set the first ripe fruit 8 days earlier than MM (*ear2.1*), whereas, IL_11-4 produced the first ripe fruit 8–13 days after MM (*ear11.1*). IL_10-1 and IL_10-2 had fruits with dark GSs at the breaker stage (*gs10.1*).

#### Fruit Size

**FIGURE 1:**
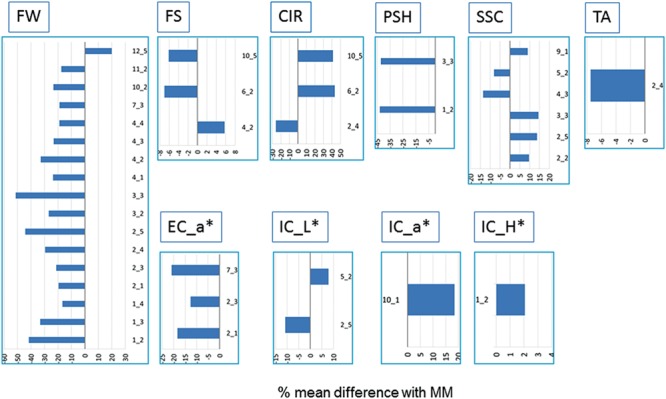
**Differences between IL means and Moneymaker (MM) across the three locations expressed in percentage (%) from the MM mean.** Only ILs showing significant mean differences with MM at *p* < 0.05 according to the Dunnet’s test in at least two locations are shown. Trait abbreviators are: fruit weight (FW), shape (FS), circular shape (CIR), shoulder height (PSH), soluble solid content (SSC), titrable acidity (TA) and internal and external color (EC; ICOL, ECOL) based on CIELab color system variables a^∗^, b^∗^, L^∗^, H, C^∗^.

The average FW of the whole IL collection across trials was 11.3% lower than MM (around 98 g per fruit, **Table 1**). The range was between 47.81 and 117.86 g (SP_3-3 and SP_12-5, respectively). FW was strongly correlated with its trait components: fruit length (FL) and fruit diameter (FD; *r* > 0.5, Supplementary Table S3). The effect of the genotype (G) was 35% of total variance; whereas the effects of the location (L) and the interaction (G-x-L) were lower than G effects, 19 and 5%, respectively (**Table 1**). FW was the trait with higher heritability (h^2^ range: 0.62–0.45, Supplementary Table S4). As a general rule, introgressions from SP had a negative effect on FW, except for SP_12-5, which increased FW 20% (**Figure 1**; Supplementary Figure S2). Three ILs (SP_1-2, SP_2-5, and SP_3-3) showed the most consistent effects among locations, and, on average, reduced FW by up to more than 40% in some locations. Fourteen additional ILs with consistent effects on FW allowed us to define a total of 12 QTLs: *fw1.1, fw1.2, fw2.1, fw2.2, fw3.1, fw3.2, fw4.1, fw4.2, fw7.1, fw10.1, fw11.1*, and *fw12.1* (**Table 2**).

**Table 1:** Trait mean values [fruit weight (FW), diameter (FD), length (FL), shape (FS), circular shape (CIR), shoulder height (PSH), soluble solid content (SSC), pH (PH), titrable acidity (TA)] and the internal and external CIELab color system variables L^∗^, a^∗^, b^∗^, C^∗^ and H for the whole Introgression Line (IL) collection and the recurrent parent Moneymaker (MM).

^∗^	Trait acronyms	Means		Effects (%)		
		ILs	MM	Genotype	Location	Genotype-x-location
Size	FW (g)	86.23	98.18	35^∗∗^	19^∗∗^	5^∗∗^
	FD (cm)	5.71	5.99	30^∗∗^	26^∗∗^	5^∗^
	FL (cm)	5.05	5.29	35^∗∗^	20^∗∗^	5^∗^
Shape	FS	0.89	0.88	23^∗∗^	9^∗∗^	9^∗∗^
	CIR	0.06	0.06	26^∗∗^	15^∗∗^	Ns
	PSH	0.04	0.04	6^∗∗^	57^∗∗^	4^∗∗^
Organoleptic	SSC (°Brix)	4.48	4.45	29^∗∗^	9^∗∗^	9^∗∗^
	PH	4.23	4.42	7^∗∗^	58^∗∗^	3^∗∗^
	TA (% citric acid)	0.29	0.29	12^∗∗^	44^∗∗^	5^∗∗^
External color	L	40.06	40.04	6^∗∗^	59^∗∗^	4^∗∗^
	a	22.22	22.45	20^∗∗^	37^∗∗^	8^∗∗^
	b	23.76	23.81	15^∗∗^	24^∗∗^	8^∗∗^
	C	32.71	32.98	16^∗∗^	38^∗∗^	7^∗∗^
	H	46.85	46.71	22^∗∗^	7^∗∗^	12^∗∗^
Internal color	L	39.2	39.52	11^∗∗^	26^∗∗^	12^∗∗^
	a	24.72	24.16	7^∗∗^	50^∗∗^	7^∗∗^
	b	27.41	27.5	14^∗∗^	1^∗∗^	13^∗∗^
	C	37.08	36.81	10^∗∗^	37^∗∗^	9^∗∗^
	H	48.42	49.07	7^∗∗^	42^∗∗^	7^∗∗^

*The genetic (G) and location (L) effects and their interaction (G-x-L) were estimated by two-way ANOVA and expressed as a percentage (%) of the total variance, with ns being non-significant, and ^∗^*p* < 0.05 and ^∗∗^*p* < 0.001, respectively.*

**Table 2:** Summary of the quantitative trait loci (QTLs) detected in the population of introgression lines for the analyzed traits [FW, FS, CIR, PSH, soluble solid content (SSC), TA, external (EC) and internal (IC) color the last two are based on CIELab color system variables L^∗^, a^∗^, b^∗^, C^∗^, H].

Trait	QTL	Chr	QTL genetic position (cM)	QTL physical position (Mb)	Localities detected	Differences with MM (%)
FW	*fw1.1*	1	57–75	74–78	3	-25
	*fw1.2*	1	77–128	78–90	2	-17
	*fw2.1*	2	47–69	38–40	2	-25
	*fw2.2*	2	69–138	42–50	3	-19
	*fw3.1*	3	51–111	7–58	3	-27
	*fw3.2*	3	111–124	58–62	2	-24
	*fw4.1*	4	0–30	0–4	2	-13
	*fw4.2*	4	30–135	4–62	2	-18
	*fw7.1*	7	39–77	60–64	2	-19
	*fw10.1*	10	0–84	0–61	2	-23
	*fw11.1*	11	69–93	49–50	2	-17
	*fw12.1*	12	94–133	62–65	2	20
CIR	*cir2.1*	2	69–86	36–42	2	-26
	*cir6.1*	6	17–56	3–35	3	43
	*cir10.1*	10	104–115	64–65	3	41
FS	*fs4.1*	4	41–94	3–35	2	6
	*fs6.1*	6	17–56	3–35	3	-7
	*fs10.1*	10	104–115	64–65	2	-6
PSH	*psh1.1*	1	50–135	3.8–70	3	-43
	*psh3.1*	3	11–124	58–62	3	-42
SSC	*ssc2.1*	2	46–63	0–36	2	10
	*ssc2.2*	2	86–139	42–49	2	14
	*ssc3.1*	3	11–124	58–62	3	14
	*ssc4.1*	4	41–94	4–57	2	-13
	*ssc5.1*	5	4–32	0–0.5	2	-8
	*ssc9.1*	9	13–92	4–57	2	9
TA	*ta2.1*	2	46–63	36–42	2	-16
EC	*ec_a^∗^2.1*	2	1–46	28–35	2	-15
	*ec_a^∗^7.1*	7	77–100	60–63	2	-20
IC	*ic_L^∗^2.1*	2	63–86	42–50	2	-11
	*ic_L^∗^5.1*	5	4–84	0–0.5	2	8
	*ic_a^∗^10.1*	10	27–31	0–2.8	2	18
	*ic_H1.1*	1	50–135	5–78	2	2

*The intervals of the QTL positions are listed in genetic and physical units following Barrantes et al. (2014). The number of locations where the QTL was significant is also indicated. The effect of the QTLs is shown as the mean difference between the locations of ILs with significant effects and recurrent parent Moneymaker (MM), expressed as a percentage with respect to MM.*

#### Fruit Shape-Related Traits

**FIGURE 2:**
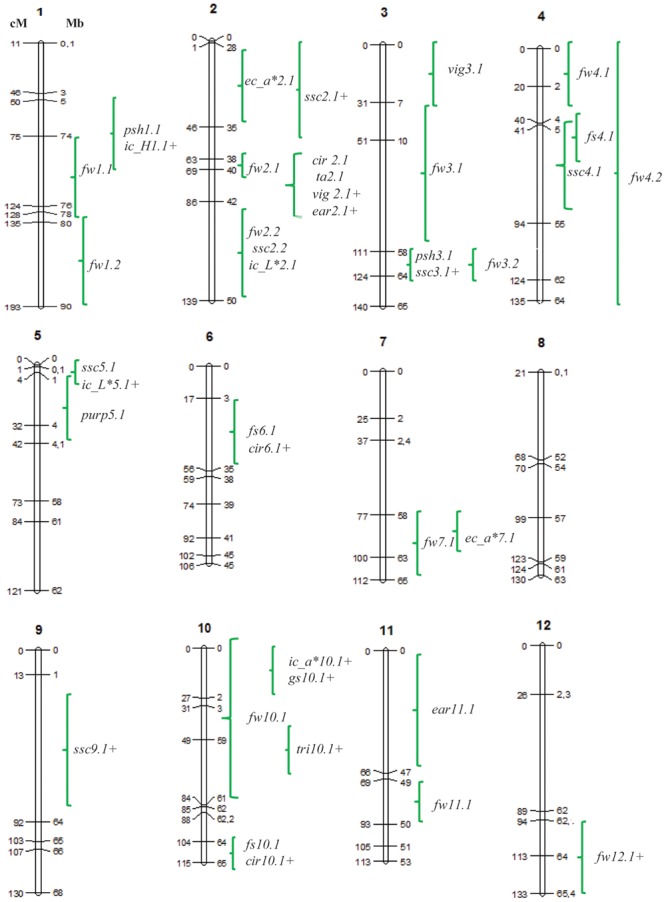
**Quantitative trait loci (QTL) positions on the tomato map.** Genetic distances (cM) are shown on the left of the chromosome drawings, and physical distances (Mb), according to the tomato genome version SL2.40, on the right. QTLs are named using an abbreviation of the trait: plant vigour (VIG), earliness (EA), presence or absence of anthocyanin coloration in branches and stems (PURP); trichome density (TRI), green shoulder on the fruit (GS), FW, FS, circular shape (CIR), shoulder height (PSH), soluble solid content (SSC), TA and internal and EC (ICOL, ECOL, respectively) based on CIELab color system variables a^∗^, b^∗^, L^∗^, H, C^∗^, followed by the chromosome on which they map and a digit indicating the number of the QTL within the chromosome. (^∗^) Indicates that the TO-937 wild allele increases the trait value.

The fruit shape index (FS) showed a relatively modest variability in the IL collection, as the fruits were nearly round, just like MM (both the IL population and MM had the same mean value of FS = 0.89), ranging from 0.82 (SP_6-2) to 0.93 (SP_4-2), being the G effect 23% of total variance (**Table 1**). FS was negatively correlated with FD (*r* = -0.35, Supplementary Table S3) and positively with FL, but to a lesser extent (*r* = 0.27), indicating that FD is the most important determinant of the variation in FS in this population. Heritability ranged from 0.48 to 0.29 (Supplementary Table S4). SP_6-2, SP_4-2, and SP_10-5 showed consistent effects on FS. Three QTLs were defined with opposite effects: *fs6.1* and *fs10.1*, which induced flattened fruits, and *fs4.1*, which induced more elongated fruits (**Figure 2**; **Table 2**).

The variability of circular fruit shape (CIR) was also low, being G effect 26% of total variance (**Table 1**), with an average value of 0.06 for the IL collection (the same value as MM, **Table 1**), ranging from 0.04 to 0.08 (SP_2-5 and SP_10-5, respectively). CIR clearly showed a high correlation (Supplementary Table S3) with FD (*r* = 0.48) but not with FL (*r* = -0.02), which supports the previous observation that FD is the principal determinant of FS variability in this population. Heritability was similar to FS, ranging from 0.47 to 0.35 (Supplementary Table S4). Additionally, SP_6-2 also increased CIR, making a total of three QTLs: *cir2.1* induced up to 26% rounder fruit, while *cir6.1* and *cir10.1* induced 42% less round fruit on average (**Figure 2**; **Table 2**).

PSH averages were the same for both the ILs and MM (0.06), with a very low G effect and the L effect being the most important one, 6 and 27% of total variance, respectively (**Table 1**). Heritability was also a little bit lower than previous fruit shape traits, ranging from 0.30 to 0.35 (Supplementary Table S4). Despite the low G effect, ILs SP_1-2 and SP_3-3 showed statistically significant differences as compared to MM in all three locations, reducing PSH by as much as 42% (**Table 2**). Two stable QTLs were defined as a result: *psh1.1* and *psh3.1* (**Figure 2**).

#### Organoleptic Related Characters

The average SSC in the IL collection was similar to that of MM (4.48 ° Brix), although the range was wide, from 3.85° to 5.1 Brix° (SP_4-3 and SP_10-6, respectively). The G effect was 29% of total variance, whereas L effect and G-x-L interaction represented 9% of total variance both of them (**Table 1**). Interestingly, correlations between SSC and fruit size and other fruit morphology-related traits were very low and non-significant (*r* < 0.1, Supplementary Table S3). Heritability was near 0.5 (Supplementary Table S4). SP_3-3 increased SSC in all three trials, whereas four additional ILs increased it in two trials (**Figure 1**; **Table 2**). On the other hand, SP_4-3 and SP_5-2 decreased SSC by 13 and 8%, respectively. A total of six consistent QTLs were defined with opposite effects: *ssc2.1, ssc2.2, ssc3.1*, and *ssc9.1* increased SSC by up to 13%, whereas *ssc4.1* and *ssc5.1* decreased SSC by up to 12% (**Table 2**; **Figure 2**).

The average TA and PH of the fruits of the IL collection were similar to those of MM (0.29 and 4.6, respectively). For both traits, the G effect was 12%, whereas L effect was the most important (44%, **Table 1**), indicating a low genetic variability for this trait in the current population. Correlations with other traits were generally low, except for SSC, which was relatively important (*r* = 0.44, Supplementary Table S3). Heritability ranged from 0.2 to 0.48 (Supplementary Table S4). Only SP_2-4 showed a significant TA decrease of 17% compared with MM in all three trials, defining the QTL *ta2.1* on chromosome 2 (**Figures 1** and **2**; **Table 2**).

#### Fruit Color

External color average values were very similar between the ILs and MM. The G effect was low for L^∗^, b^∗^ and C ^∗^ (6–16%), moderate for a^∗^ and H (>20%, **Table 1**), and the L effect was important in most of them (>24% of total variance, *p* < 0.001). Meanwhile, interaction G-x-L was generally lower than 12% of total variance. EC positively correlated moderately with fruit size (*r*∼0.2, *p* = 0.004, Supplementary Table S3) and negatively with the organoleptic traits SSC and TA (*r* < 0.4, *p* = 0.004). Heritability ranged from 0.23 to 0.55 (Supplementary Table S4). Three ILs, SP_2-1, SP_2-3 and SP_7-3, showed significant differences in the a^∗^ color component, defining the QTLs *ec_a^∗^2.1* and *ec_a^∗^7.1* (**Figure 1**), associated in both cases with a reduction of the color red (**Figure 2**; **Table 2**).

Internal color average values were very similar between the ILs and MM, except for the components C^∗^ and H, which were slightly higher or slightly lower in the IL collection, respectively. The G effects represented between 7 and 14 of the total variance, being the L effect was higher in most cases (>26%), with the exception of b^∗^ (**Table 1**). The interaction G-x-L for IC components ranged from 7 to 13% of total variance (**Table 1**). No high correlation values between IC and the other traits were detected (Supplementary Table S3). ILs SP_1-2, SP_5-2, SP_10-2 increased the H, L^∗^ and a^∗^ components, respectively, whereas SP_2-5 decreased L^∗^ (**Figure 1**). Heritability ranged from 0.23 to 0.44 (Supplementary Table S4). A total of four QTLs were defined: *ic_a^∗^2.1* and *ic_a^∗^10.1*, associated with an increased red-colored fruit, and *ic_H1.1* and *ic_L^∗^5.1*, which reduced red coloration (**Figure 2**; **Table 2**).

In summary, a total of 33 QTLs with consistent effects in at least two locations were defined (**Figure 2**). These QTLs were mapped and covered different regions in all chromosomes, except chromosome 8, where no QTL was detected. The distribution of QTLs was not homogeneous among chromosomes, with the largest number of QTLs (8) being found in chromosome 2. Chromosomes 1, 3, 4, and 10 had four QTLs and the rest of the chromosomes showed fewer QTLs.

## Discussion

### QTLs Detected in the IL Population

To the best of our knowledge, the current work reports the first thoroughly evaluated IL collection with an *S. pimpinellifolium* accession as donor. SP is the phylogenetically closest wild species to the cultivated tomato, which could explain why, in general, the phenotypes of the ILs were so similar to MM, especially compared with other IL collections derived from more distant wild species (Eshed and Zamir, 1995; Bernacchi et al., 1998; Monforte et al., 2001). As a result, deleterious linkage drag effects are minimized in this collection, which facilitates the exploitation of this genetic resource in plant breeding. Furthermore, MM is a fresh-market variety, in contrast to the processing cultivars more widely used as genetic background (Eshed and Zamir, 1995; Monforte and Tanksley, 2000a).

A few extreme phenotypes were observed in the population, some of which affected general plant growth and architecture. SP_3-1 showed severe plant growth impairment, limiting fruit production drastically. This phenotype is very similar to the one caused by the *pauper* mutation, which maps on the short arm of chromosome 3 (tgrc.ucdavis.edu), so *vig3.1* could represent an allele of the *pau* gene. As neither MM nor TO-937 showed any growth impairment, it is unlikely that a new mutation on *pau* would have occurred during IL development. We think that the effect can most likely be attributed to interactions between TO-937 alleles (*pau* or other genes) within the SP_3-1 introgression and other genes in MM genetic background. Another classical mutant, *pro* or *procer*a (tgrc.ucdavis.edu), which exhibits a more rapid growth rate, produces tall, slender, weak plants and elongated internodes, and co-locates with the earliness QTL *ear11.1* in IL SP_11-4.

SP_5-2 and SP_5-3 displayed the color purple on both primary and axillary shoots, indicating anthocyanin over-accumulation. The *Af* gene (*anthocyanin free*, tgrc.ucdavis.edu) is involved in the accumulation of anthocyanins as mutations (*af*) in this gene, and produces plants that do not accumulate anthocyanin in all tissues (Burdick, 1958). This gene is located in the short arm of chromosome 5 and encodes a chalcone isomerase enzyme (CHI) that catalyzes the synthesis of 2(*S*)-naringenin, a key intermediate in the flavonoid pathway, which is required for flavonoid production (Kang et al., 2014) This means that *purp5.1* may be allelic to *Af*. As in the previous example, neither MM nor TO-937 displayed anthocyanin accumulation under normal non-stress conditions, suggesting that epistatic interactions between TO-937 alleles at CHI and with MM genetic background likely induced an expression of CHI, which would explain the resulting anthocyanin accumulation.

SP_10-3 displayed a much higher trichome density than MM, with long type-I trichomes (Luckwill, 1943) densely covering leaves and stems. Yang et al. (2011) isolated the *Woolly* gene (*Wo*), essential for trichome formation in all vegetative parts. This gene is located in the long arm of chromosome 2, meaning that SP_10-3 most likely contains a novel gene involved in trichome development. Trichomes have been associated with insect resistance (Guo et al., 1993; Blauth et al., 1998; Maluf et al., 2010). Salinas et al. (2013) mapped two QTLs in chromosome 2 from the wild tomato TO-937 that control resistance against the two-spotted spider mite (*Tetrany chusurticae* Koch) and which is based on short, glandular, type-IV trichomes that produce acylsucroses (Alba et al., 2009). However, no gene or QTL involved in insect resistance has been mapped in chromosome 10 so far, which suggests that the increase of trichome density induced by *tric10.1* is not related to disease resistance. Since the *h* gene (hairs absent, tgrc.ucdavis.edu) produces absence of long trichomes (except on hypocotyl) and is located in the long arm of chromosome 10, it is likely that *tric10.1* is allelic to *h.* No candidate gene is known for this gene.

Regarding fruit quality traits, in general no important deleterious phenotypes were observed. SP_10-1 and SP_10-2 yielded dark green-shouldered fruits as a consequence of the allelic replacement of the *uniform ripening* (*u)* gene that is located in the introgressions presented by the ILs and which corresponds to the reconstitution of the function of the *GLK-2* gene involved in chloroplast development in fruit (Powell et al., 2012).

Fruit weight showed the highest genetic variability and heritability as well as the largest number of detected QTLs. The striking phenotypic differences between parental lines explain these results. The increase in FW is an important domestication and diversification trait. Major QTLs involved in this trait have been isolated in the last few years (Monforte et al., 2014), and some QTLs detected in the current report provide additional evidence for them: *fw2.2* (Frary et al., 2000), *fw3.2* (Chakrabarti et al., 2013) FAB, FIN (Xu et al., 2015), which are most likely responsible for the FW QTLs reported herein in chromosomes 2, 3, 4, and 11, respectively. These QTLs are involved in either domestication or improvement (Lin et al., 2014), together with other loci in chromosomes 5, 7, 9, and 12. The current population revealed additional FW QTLs on chromosomes 1 (SP_1-2, SP1_3, and SP_1-4) and 10 (SP_10-2), with effects of a comparable magnitude (**Figure 1**) to those previously described, even though they have not been previously associated to either domestication or improvement. These QTLs have not been found in other mapping populations derived from SP accessions, although they may be orthologous to FW QTLs from other wild tomato species, such as *S. pennellii* (Eshed and Zamir, 1995). Also worthy of note are the transgressive effects of IL_12-5 that increase FW. QTLs involved in FW have been mapped in this region, either increasing (Causse et al., 2004) or decreasing (Fulton et al., 2000; Prudent et al., 2009; Xu et al., 2013), indicating that there must be allelic variability for this QTL in the wild species germplasm that was probably not selected during domestication into the cultivated gene pool.

With respect to FS, the genetic variability observed in the current IL population was modest. At the same time, the number of detected QTLs was small and their effects were low. The QTL *cir2.1*, responsible for 26% of fruit round shape variability likely corresponds to QTLs detected in previous SP-derived populations (Grandillo and Tanksley, 1996; Tanksley et al., 1996; Bernacchi et al., 1998; van der Knaap and Tanksley, 2003). The physical position coincides with *ovate*, so *cir2.1* could be a weak allele of the ovate gene (Liu et al., 2002). QTLs *cir6.1* and *cir 10.1*, mapped in chromosomal regions previously associated with FS on chromosomes 6 (Bernacchi et al., 1998; van der Knaap and Tanksley, 2003) and 10 (Chen et al., 1999) in SP-derived populations, are most likely allelic QTLs.

Shoulder height QTLs were mapped for the first time by Brewer et al. (2007) in chromosomes 1, 2, and 7. In the current study, *psh1.1* and *psh3.1* were mapped, with *psh1.1* located at the same position previously defined by Brewer et al. (2007) on chromosome 1. The appearance of shoulder height in tomato fruit is restricted to cultivated tomato, which is probably a consequence of domestication. It seems unlikely that ancient farmers would have selected for this trait intentionally. One possible explanation is a pleiotropic effect of fruit size increase; in fact, SP_1-2 and SP_3-3 decreased both FW and PSH, although other ILs that reduced FW did not show any effects on PSH. An alternative explanation is that the pleiotropic effect could be QTL-specific. SSC is one of the primary quality traits of tomato fruits. The amount of genetic variability in the current population was higher than it was for FS, with a total of five QTLs with opposite effects being detected (three increasing, two decreasing). No correlation was found between SSC and FW. SP_2-2 and SP_9-1 increased SSC but did not decrease FW, making those QTLs an appropriate choice for increasing SSC without negative effects on FW. QTLs on ILs SP_3-3, SP_4-3, and SP_5-1 have been detected in a very limited number of previous works (Tanksley et al., 1996; Chen et al., 1999), whereas QTLs on SP_2-2, SP_2-5, and SP_9-1 have been detected more frequently (Grandillo and Tanksley, 1996; Tanksley et al., 1996; Chen et al., 1999; Doganlar et al., 2002; Causse et al., 2004; Chaïb et al., 2006; Xu et al., 2013; Pereira da Costa et al., 2013). The QTL on SP_2-5 is likely a pleiotropic effect of *fw2.2* (Frary et al., 2000), whereas the effect on SP_9-1 could be due to the apoplastic invertase *Lin5* (Fridman et al., 2004). This difference in SSC QTL detection among different mapping populations most likely reflects a high genetic variability for this trait in both cultivated and wild germplasm.

Fruit color is also an important quality trait as it is associated with lycopene accumulation. Both EC and IC components showed a moderate genetic variance in this population, indicating low allelic diversity between MM and TO-937. Correlations between EC and IC components were low and non-significant, confirming a different genetic control for these traits (Monforte et al., 2001). All EC QTLs from TO-937 reduced red coloration, an unexpected result since TO-937 fruits are redder than MM fruits. On the other hand, SP_1-2, SP_2-5, and SP_10-1 displayed a more intense red IC, whereas SP_5-2 showed a diminished red IC, which makes those first ILs promising for the improvement of the nutritional quality of tomatoes. *Phytoene synthase 2* (*psy2*) is located in the SP_2-5 introgression (Bartley and Scolnik, 1993), and is a strong candidate gene. QTLs for IC have been detected previously in the same genomic region, which expands the introgression of SP_1-2 (Grandillo and Tanksley, 1996; Bernacchi et al., 1998; Liu et al., 2003). Since the *high pigment-2* (*hp-2*) locus maps in the same region (van Tuinen et al., 1997), it is likely that the current QTL is an allele of *hp-2* with weaker effects. The loci on SP_5-2 and SP_10-1 are the most promising ones, although the introgressions still harbor a large number of genes that may turn out to be candidate genes.

### Comparison of QTL Detection between RIL and IL Populations

A RIL population derived from the same parents as the current IL library was recently used to map QTLs involved in FW, SSC, TA and PH, among other traits (Capel et al., 2015), which gave us the opportunity to assess the effect of the genetic structure of the mapping population on QTL detection. In general, the fruits of the RIL population were smaller than those of the IL population, which was probably due to the accumulation of FW QTLs from SP in the RIL genomes. However, FW showed a similar range of phenotypic variation to the ILs in absolute values for FW (1.51–58.24 g among RILs, compared to 47.81–117.86 g among the ILs), while a higher range was observed for SSC, TA, and PH among RILs than the ILs. These differences in the range of variation suggest that additive gene action is common for FW, whereas, for the other traits, epistatic interactions among QTLs are significant contributors to the genetic variance.

Several FW QTLs were detected in both the RIL and IL populations on the same regions of chromosomes 1, 2, 7, and 11. Two additional QTLs were detected on other regions of chromosome 7 in the RILs, whereas FW QTLs on chromosomes 3, 4, 10, and 12 were only detected with the ILs. Moreover, QTLs on chromosomes 1 and 2 were separated as two linked QTLs with the ILs. Therefore, most of the QTLs detected in the RIL population were verified with the ILs, and further QTLs were also detected, which reflects a high consistency of FW QTLs across generations. This is compatible with the previous hypothesis on the additive effects of FW QTLs and the efficacy of ILs in detecting FW QTLs.

Of the seven SSC QTLs reported in the RIL population and the six SSC QTLs in the IL population, only two mapped in the same chromosomal region on chromosomes 2 and 3. Despite the lower consistence between generations found for TA, none of the TA QTLs detected in the RILs could be verified with the ILs, and the only QTL detected in the ILs (*ta2.1*) was not present in the RIL QTL map.

The stability of QTL effects over generations is a crucial issue when implementing MAS. The lack of this stability is one of the major factors that could explain the limited use of MAS for QTLs (Collard and Mackill, 2008). In tomato, what is probably the most thorough study on QTL effect stability over generations was carried out by Chaïb et al. (2006), where they found that, out of 10 QTLs detected in an RIL population, five were detected in BC3S1 and eight in BC3S3 populations, indicating a good stability.

In the current report, we have found that QTL stability depends on the trait in question, as it is high for FW, low for SSC and absent for TA. The discrepancy in QTL detection among generations can be attributed to experimental (environmental) and biological factors. Interaction with genetic background must have an important role; the fact that different traits show different levels of stability can be explained by differences in the importance of genetic background interactions in the expression of the QTLs, i.e., the prevalence of additive gene action versus epistasis. Of note is the fact that the comparison of trait distributions between the RILs and the ILs indicated that epistatic interactions most likely have an important role in SSC and TA traits, as all show a low QTL stability. Another factor could be the pleiotropic effects of fruit size on SSC and TA, as RIL fruits are much smaller than IL fruits.

The differences in QTL stability across generations observed in the current work reinforce the necessity of developing populations in the proper genetic background, depending on the objectives of the study. Transferring QTLs to different genetic backgrounds will always be a challenge, and it will probably always depend on each particular case. ILs are the populations of choice, especially for applied MAS, as the QTLs of interest can be evaluated in the final genetic background.

## Author Contributions

WB: was involved in data acquisition, analysis, interpretation of the data, and drafted the manuscript; GL-C, SG-M, AA, and JR: was involved in data acquisition and critical review of the manuscript; RF-M: was involved in the design of the work, data acquisition, and critical review of the manuscript; AG: was involved in the design of the work, data acquisition and critical review of the manuscript; AM: was involved in the design of the work, data acquisition, analysis, and drafted the manuscript. All authors approve this version of the manuscript and agree to be accountable for all aspects of the work.

## Conflict of Interest Statement

The authors declare that the research was conducted in the absence of any commercial or financial relationships that could be construed as a potential conflict of interest.

## Footnotes

1 http://oardc.ohio-state.edu/vanderknaap/
