# Supplementary material for: Exploring New Alleles Involved in Tomato Fruit Quality in an Introgression Line Library of Solanum pimpinellifolium
Source: Front Plant Sci. 2016 Aug 17;7:1172. doi: 10.3389/fpls.2016.01172 (PMC4987366; doi:10.3389/fpls.2016.01172)
Supplement: Supplementary file 4 [file Table_4.DOC]

Table S4. Heritability (h^2^=Vg/Vt, estimated by two-way ANOVA) for fruit weight (FW), diameter (FD), length (FL), shape (FS), circular shape (CIR), shoulder height (PSH), soluble solid content (SSC), pH (PH), titrable adicity (TA) and the internal and external CIELab color system variables L*, a*, b*, C* and H for Introgression Line grown in the three locations (Alginet, Orihuel and mälaga).  **NS** non significant, and (^**^) significant effects at p<0.001.

| **Traits** | **Acronyms** | **Heretability** | | |
| --- | --- | --- | --- | --- |
|  |  |  |  |  |
|  |  | **Alginet** | **Orihuela** | **Málaga** |
| **Fruit Size** | FW (g) | 0.62** | 0.52** | 0.45** |
|  | FD (cm) | 0.61** | 0.50** | 0.41** |
|  | FL (cm) | 0.62** | 0.49** | 0.49* |
| **Fruit Shape** | FS | 0.48** | 0.36** | 0.29** |
|  | CIR | 0.47** | 0.49^ns^ | 0.35** |
|  | PSH | 0.30** | 0.35** | 0.30** |
| **Organoleptic** | SSC (ºBrix) | 0.51** | 0.48** | 0.43** |
|  | PH | 0.25** | 0.40** | 0.20** |
|  | TA (% citric acid) | 0.37** | 0.34** | 0.46** |
| **External Color** | L | 0.37** | 0.26** | 0.23** |
|  | a | 0.51** | 0.55** | 0.32** |
|  | b | 0.41** | 0.29** | 0.23** |
|  | C | 0.48** | 0.46** | 0.25** |
|  | H | 0.42** | 0.37** | 0.33** |
| **Internal Color** | L* | 0.44** | 0.49** | 0.24** |
|  | a | 0.39** | 0.45** | 0.29** |
|  | b | 0.38** | 0.31** | 0.23** |
|  | C | 0.40** | 0.36** | 0.24** |
|  | H | 0.36** | 0.42** | 0.29** |
