# Supplementary material for: Exploring New Alleles Involved in Tomato Fruit Quality in an Introgression Line Library of Solanum pimpinellifolium
Source: Front Plant Sci. 2016 Aug 17;7:1172. doi: 10.3389/fpls.2016.01172 (PMC4987366; doi:10.3389/fpls.2016.01172)
Supplement: Supplementary file 5 [file Presentation_1.PPT]

## Slide 1
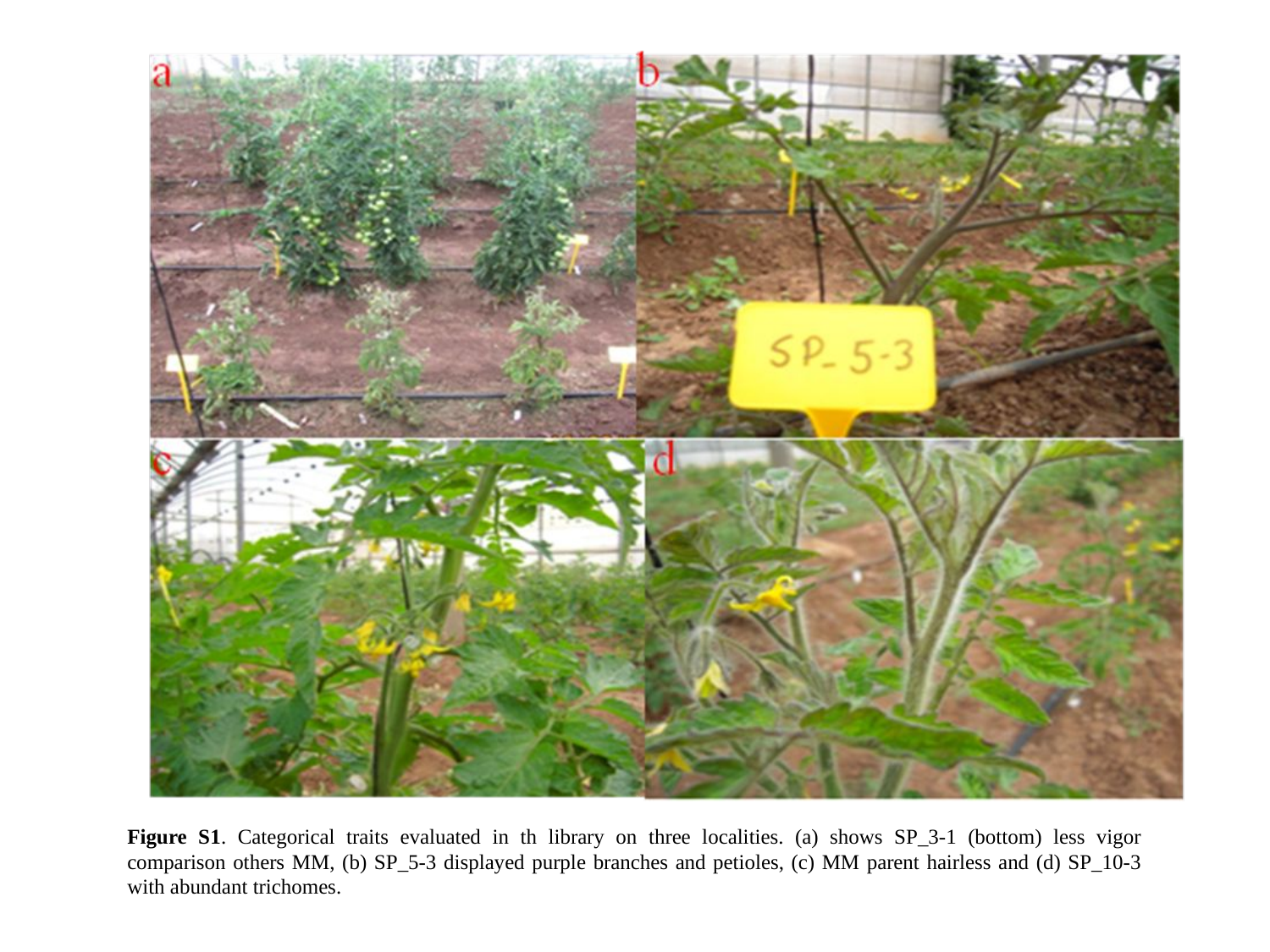

Figure S1. Categorical traits evaluated in th library on three localities. (a) shows SP_3-1 (bottom) less vigor comparison others MM, (b) SP_5-3 displayed purple branches and petioles, (c) MM parent hairless and (d) SP_10-3 with abundant trichomes.
