# Supplementary material for: Exploring New Alleles Involved in Tomato Fruit Quality in an Introgression Line Library of Solanum pimpinellifolium
Source: Front Plant Sci. 2016 Aug 17;7:1172. doi: 10.3389/fpls.2016.01172 (PMC4987366; doi:10.3389/fpls.2016.01172)
Supplement: Supplementary file 6 [file Presentation_2.PPT]

## Slide 1
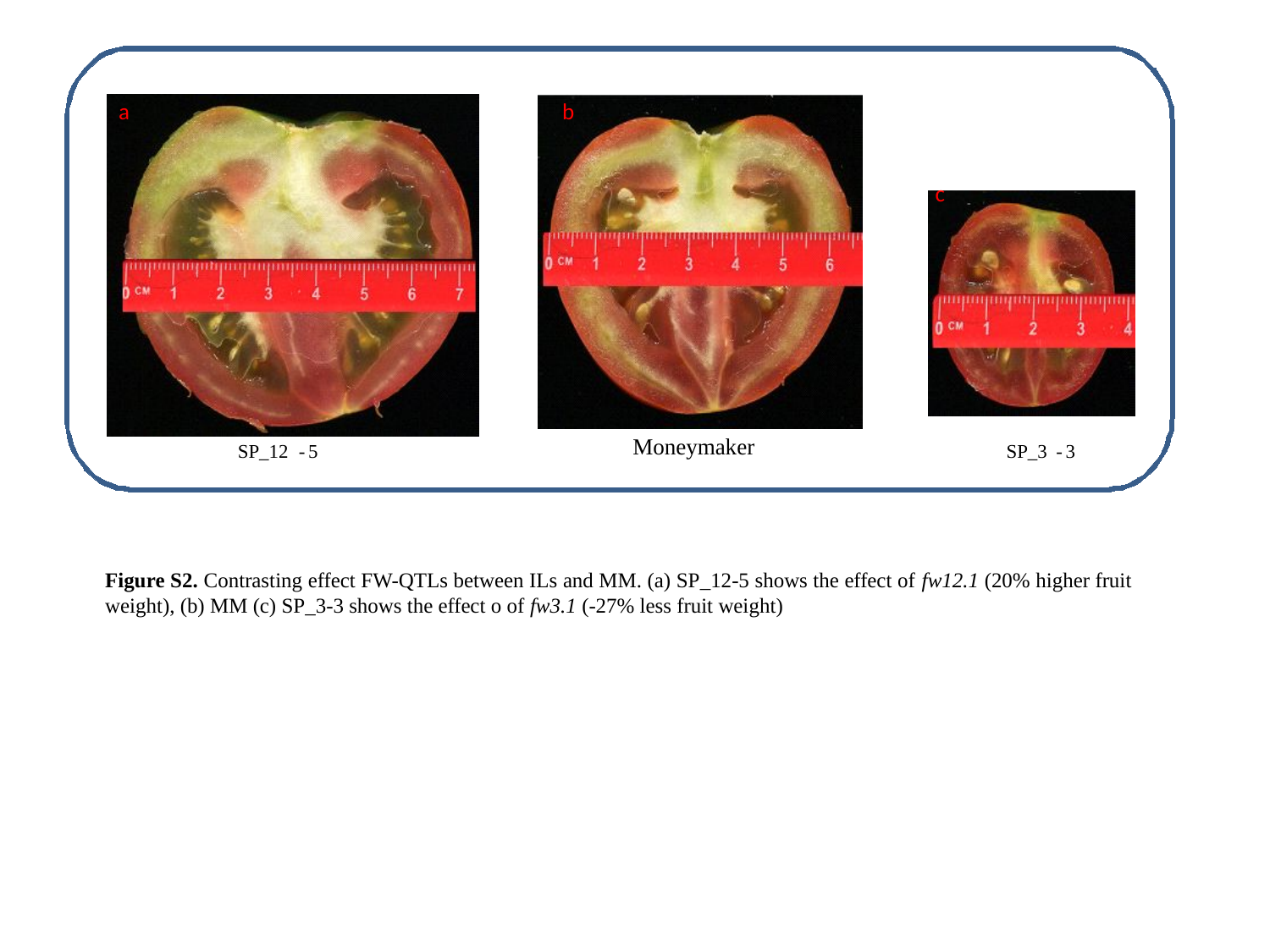

a
b
c
Moneymaker
SP_12
-
5
SP_3
-
3
Figure S2. Contrasting effect FW-QTLs between ILs and MM. (a) SP_12-5 shows the effect of fw12.1 (20% higher fruit weight), (b) MM (c) SP_3-3 shows the effect o of fw3.1 (-27% less fruit weight)
